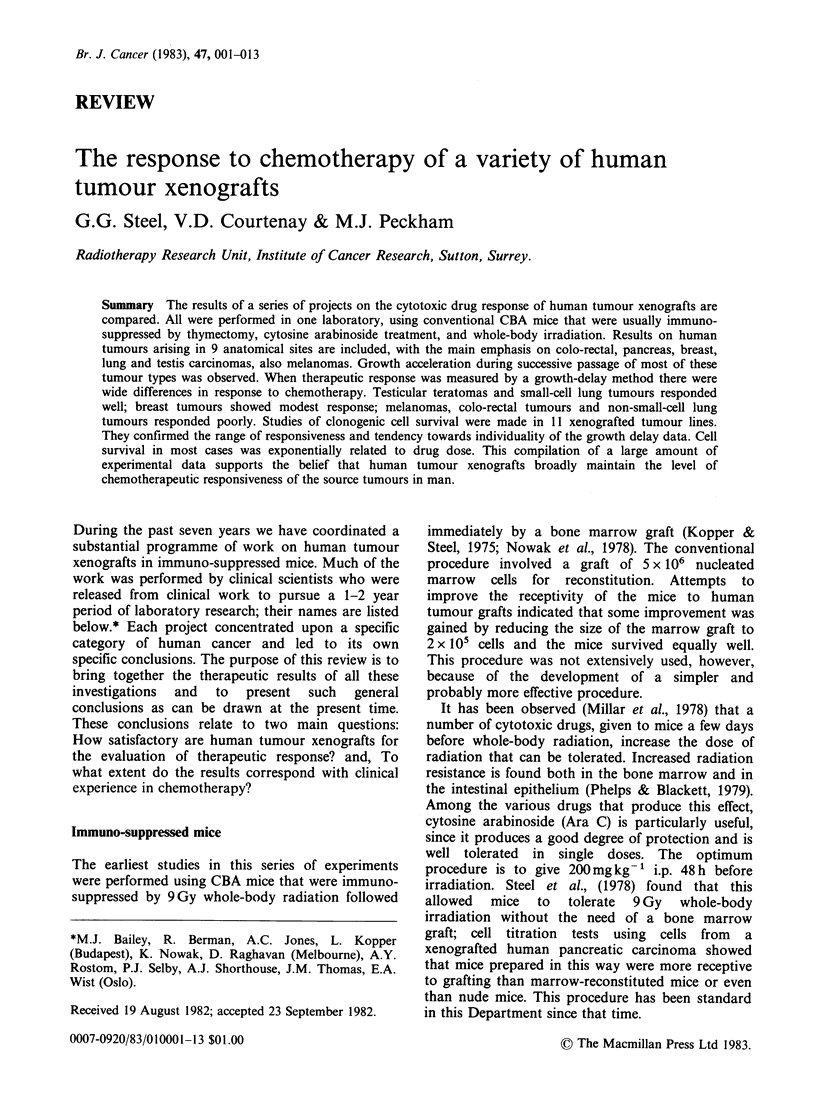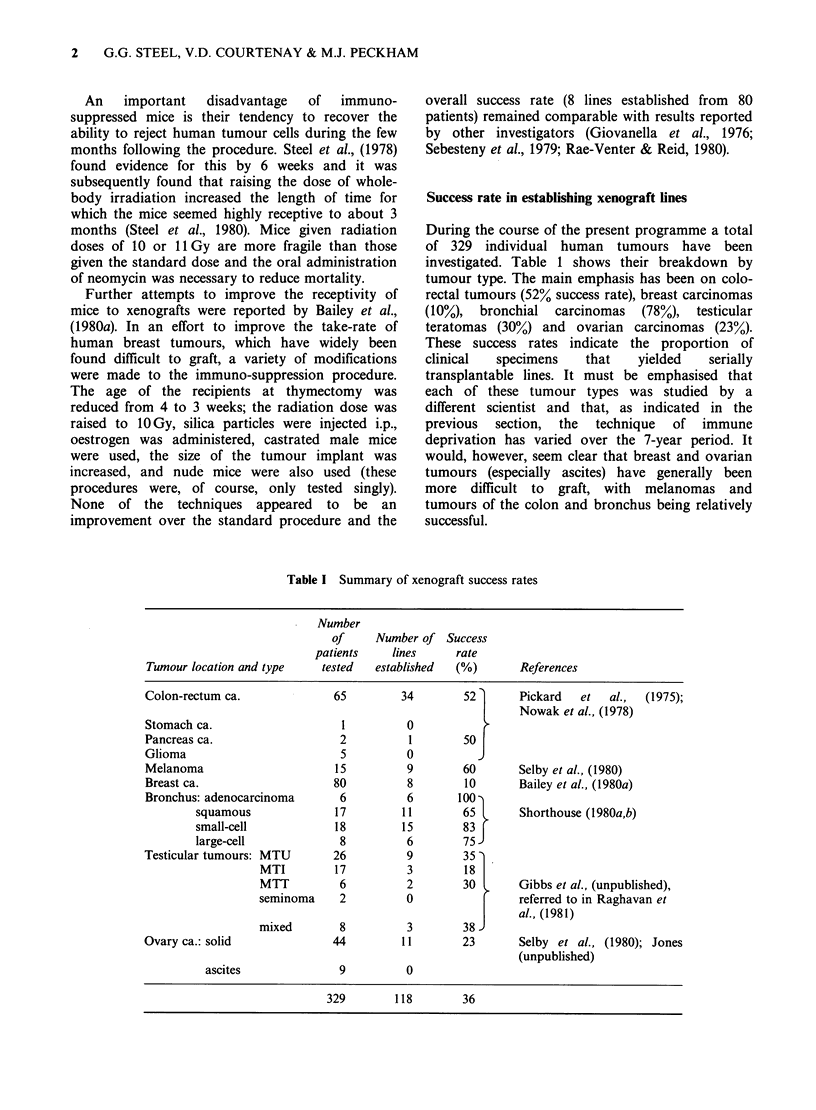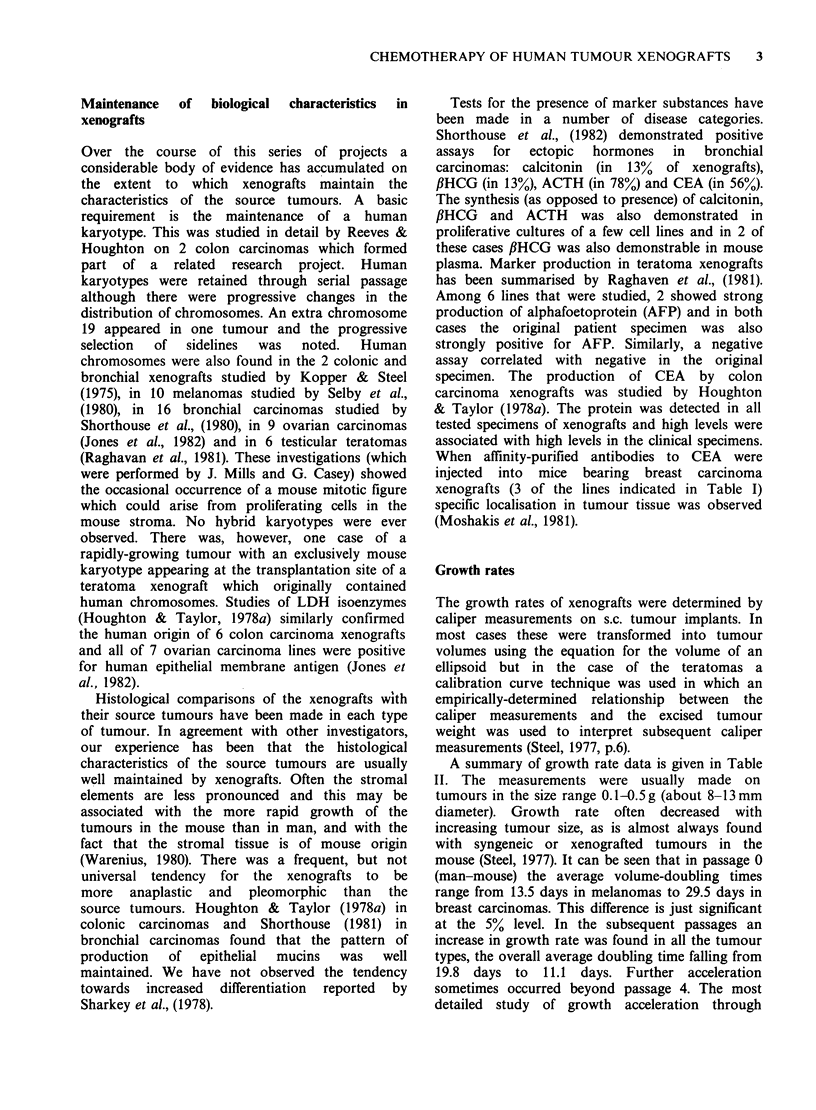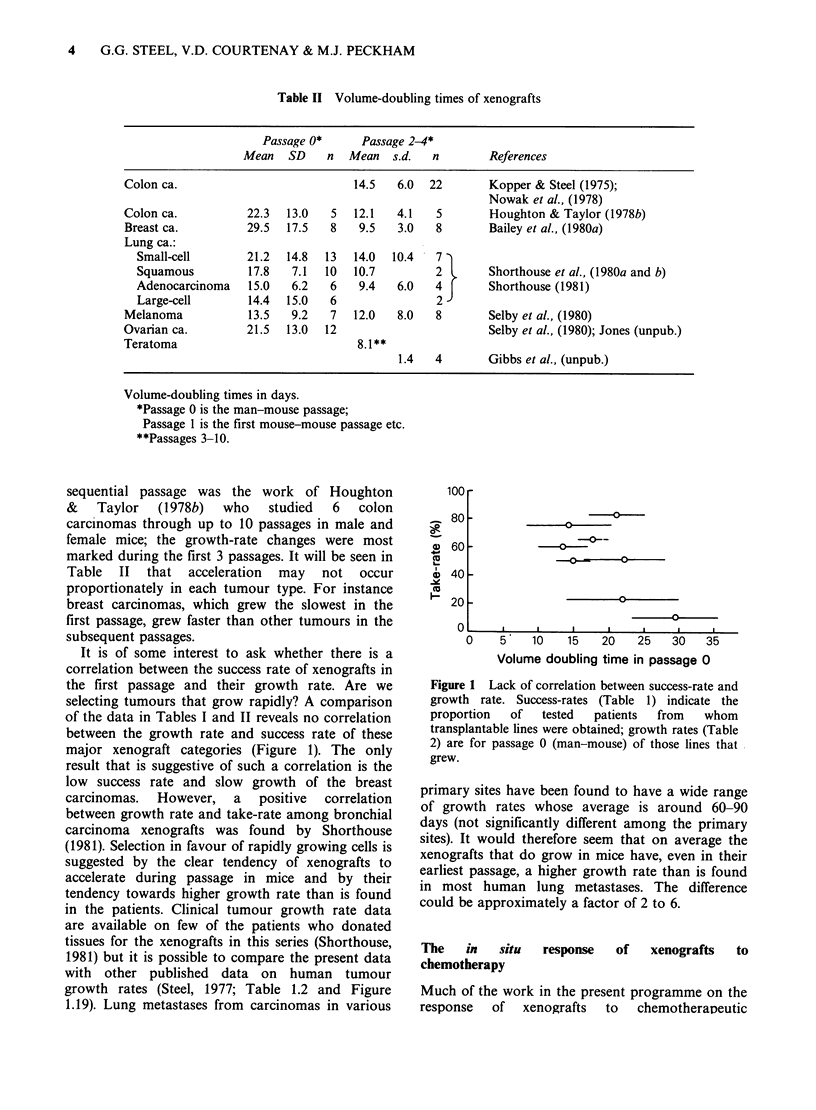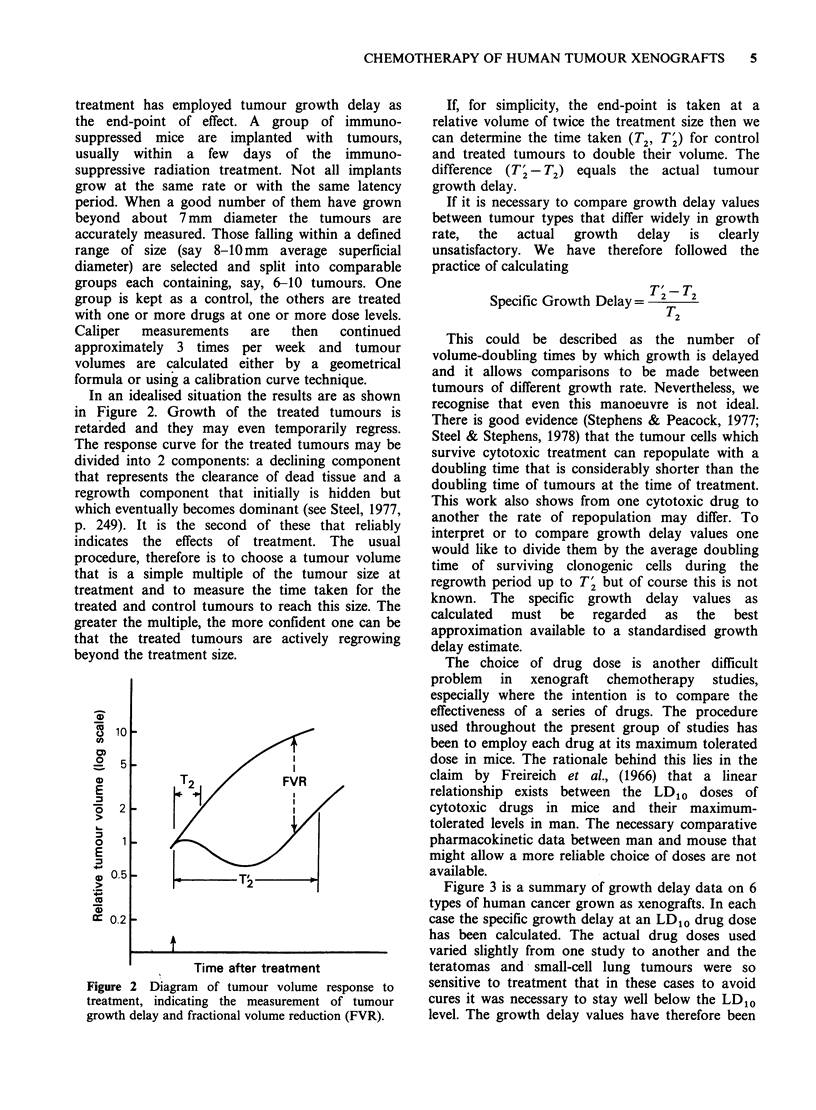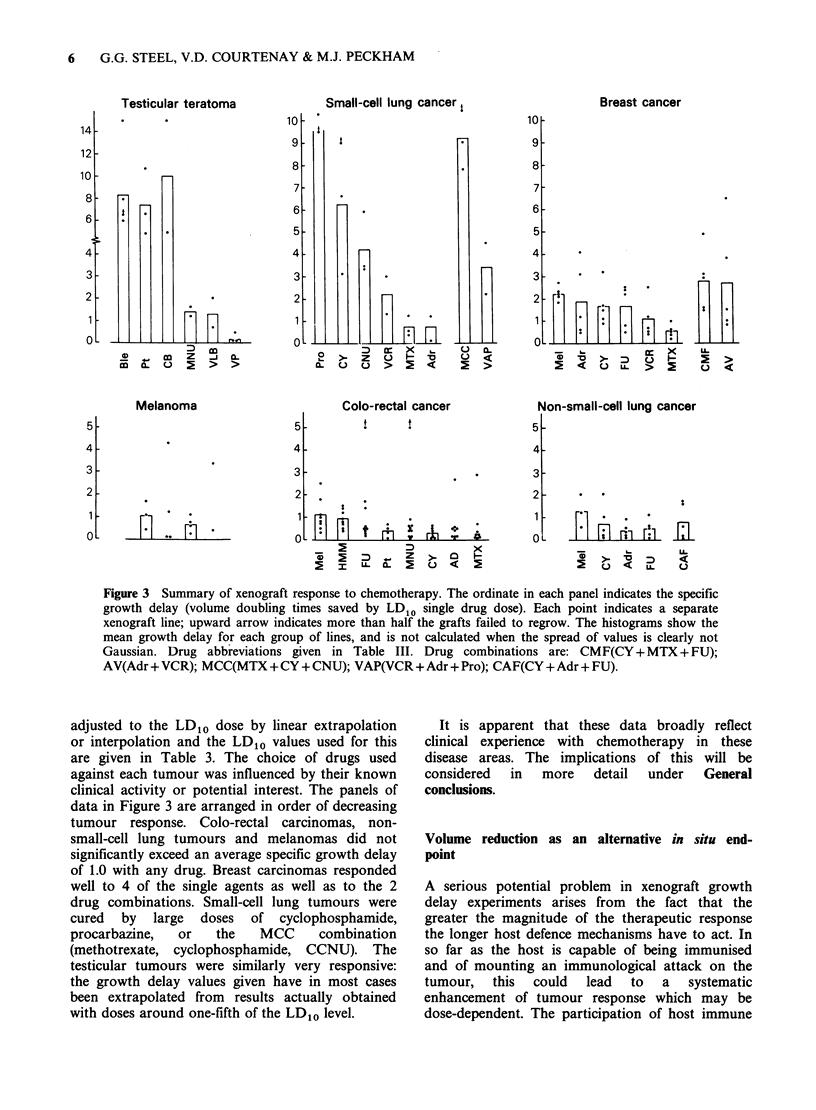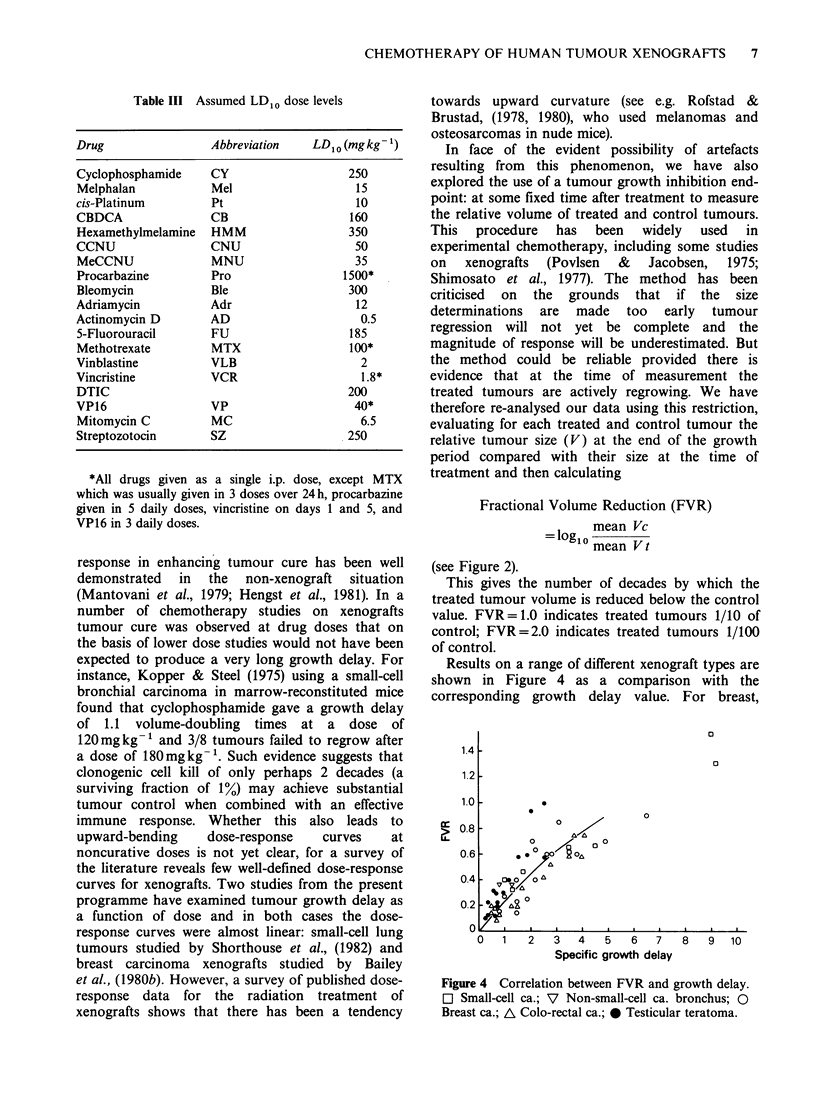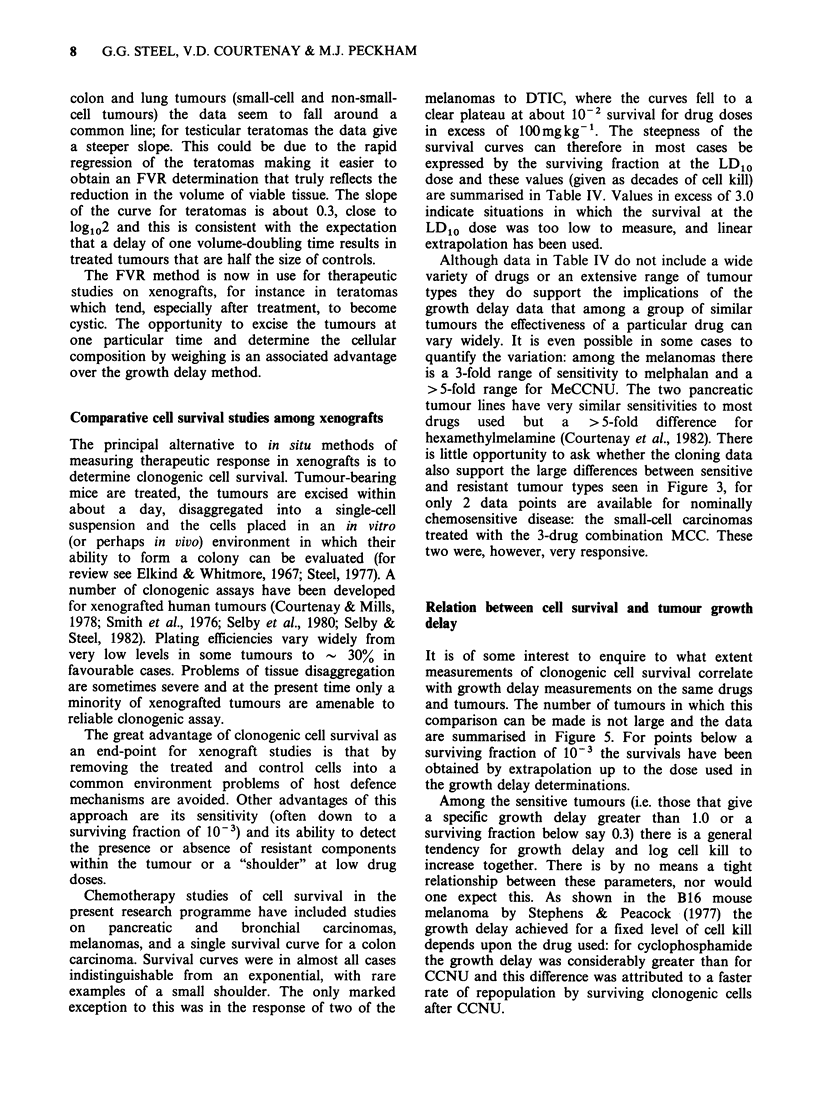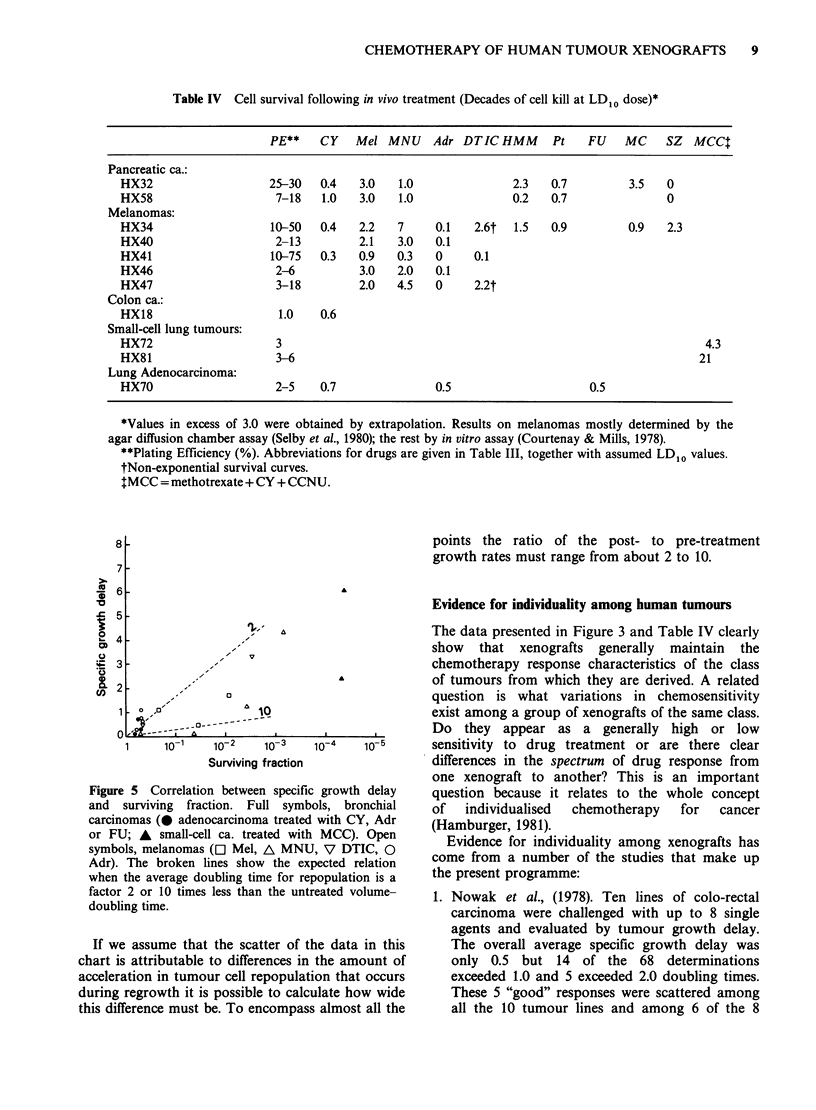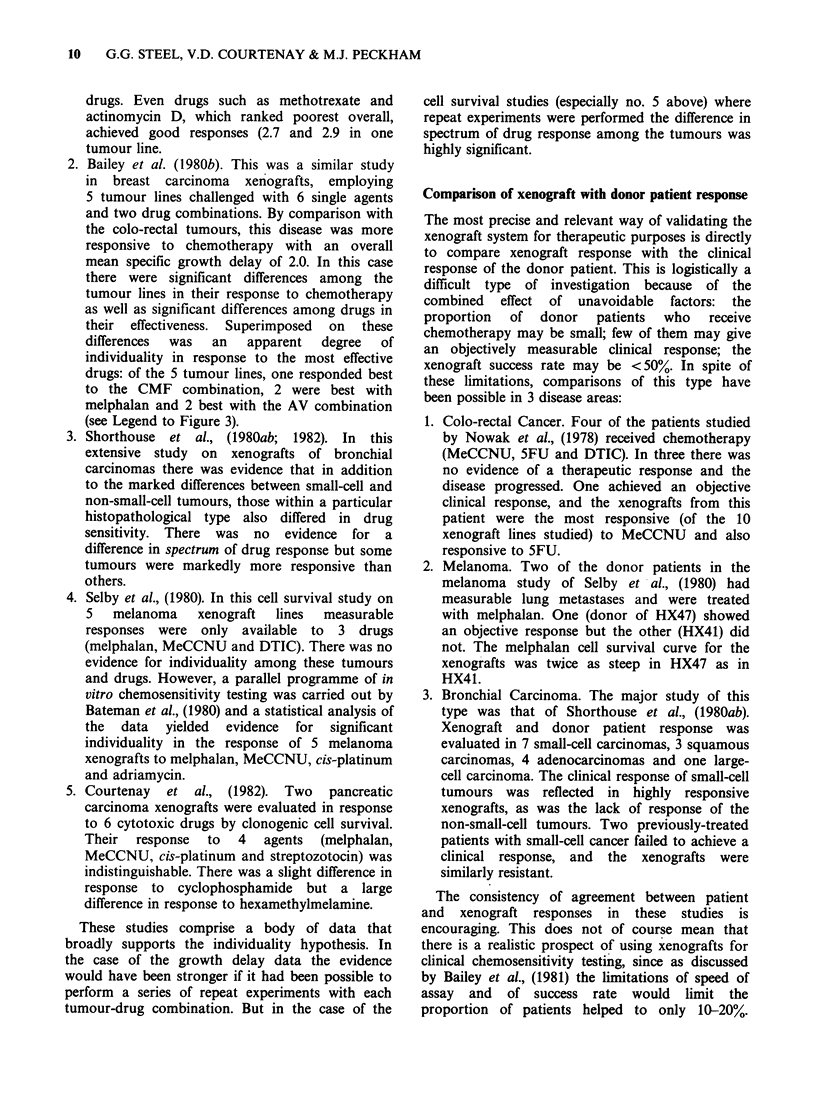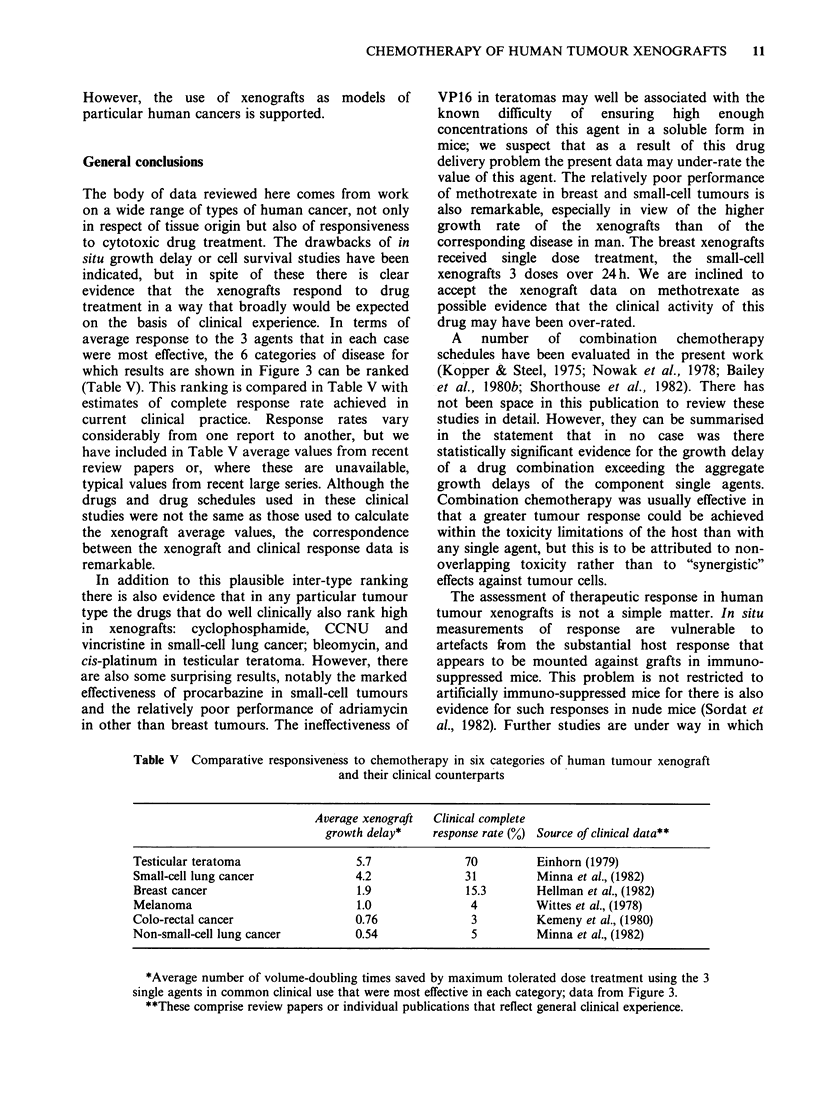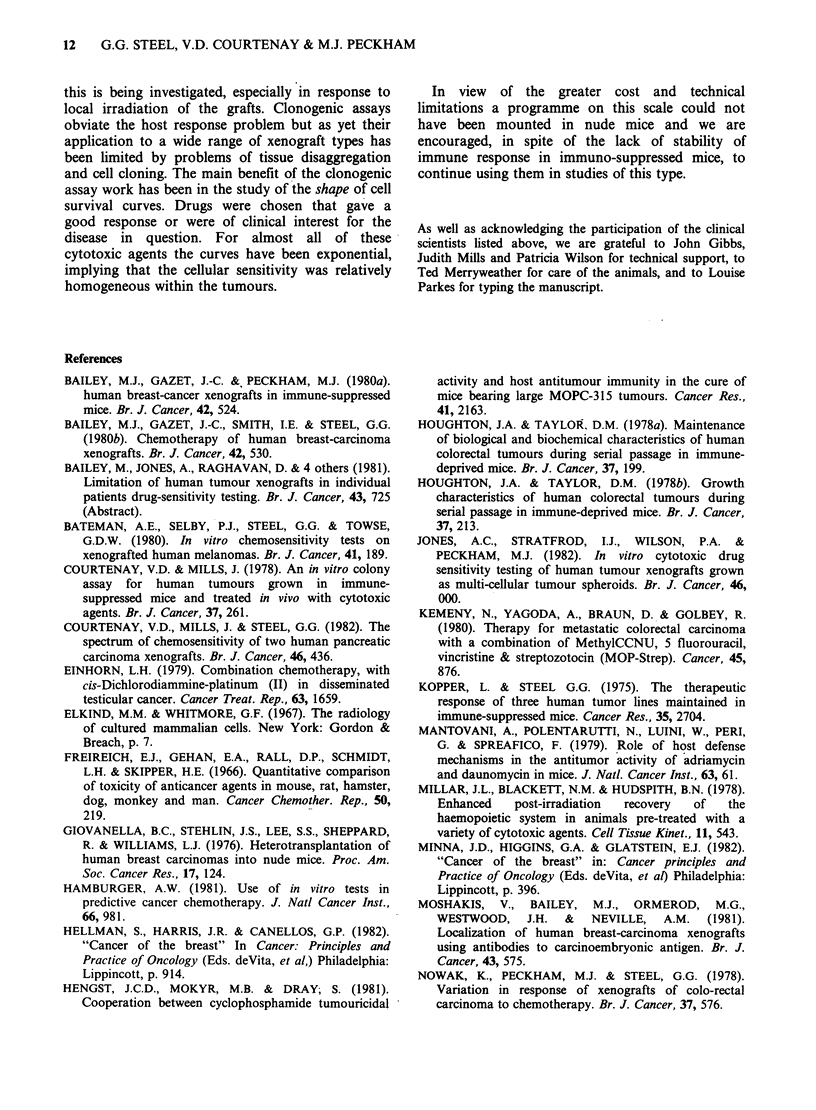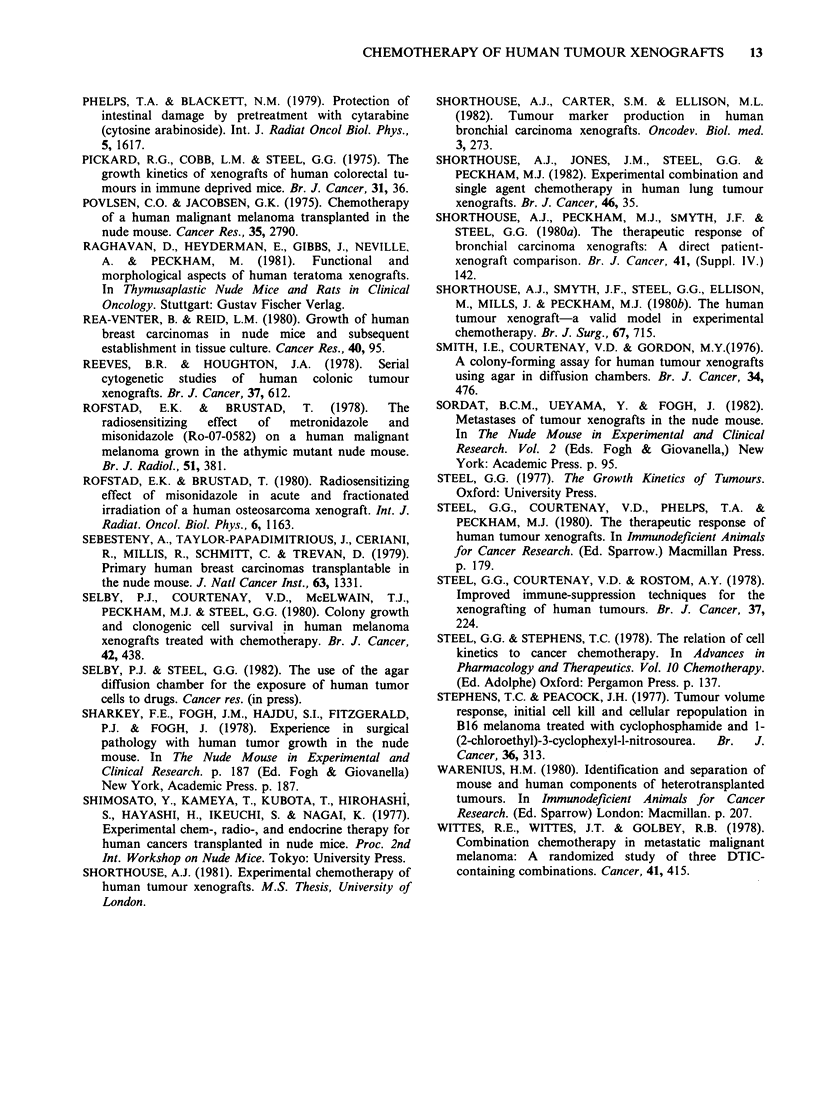# The response to chemotherapy of a variety of human tumour xenografts.

**DOI:** 10.1038/bjc.1983.1

**Published:** 1983-01

**Authors:** G. G. Steel, V. D. Courtenay, M. J. Peckham

## Abstract

The results of a series of projects on the cytotoxic drug response of human tumour xenografts are compared. All were performed in one laboratory, using conventional CBA mice that were usually immunosuppressed by thymectomy, cytosine arabinoside treatment, and whole-body irradiation. Results on human tumours arising in 9 anatomical sites are included, with the main emphasis on colo-rectal, pancreas, breast, lung and testis carcinomas, also melanomas. Growth acceleration during successive passage of most of these tumour types was observed. When therapeutic response was measured by a growth-delay method there were wide differences in response to chemotherapy. Testicular teratomas and small-cell lung tumours responded well; breast tumours showed modest response; melanomas, colo-rectal tumours and non-small-cell lung tumours responded poorly. Studies of clonogenic cell survival were made in 11 xenografted tumour lines. They confirmed the range of responsiveness and tendency towards individuality of the growth delay data. Cell survival in most cases was exponentially related to drug dose. This compilation of a large amount of experimental data supports the belief that human tumour xenografts broadly maintain the level of chemotherapeutic responsiveness of the source tumours in man.


*Br. J. Cancer* (1983), **47**, 001–013

### REVIEW

### The response to chemotherapy of a variety of human tumour xenografts

G.G. Steel, V.D. Courtenay & M.J. Peckham

*Radiotherapy Research Unit, Institute of Cancer Research, Sutton, Surrey.*

**Summary** The results of a series of projects on the cytotoxic drug response of human tumour xenografts are compared. All were performed in one laboratory, using conventional CBA mice that were usually immuno-suppressed by thymectomy, cytosine arabinoside treatment, and whole-body irradiation. Results on human tumours arising in 9 anatomical sites are included, with the main emphasis on colo-rectal, pancreas, breast, lung and testis carcinomas, also melanomas. Growth acceleration during successive passage of most of these tumour types was observed. When therapeutic response was measured by a growth-delay method there were wide differences in response to chemotherapy. Testicular teratomas and small-cell lung tumours responded well; breast tumours showed modest response; melanomas, colo-rectal tumours and non-small-cell lung tumours responded poorly. Studies of clonogenic cell survival were made in 11 xenografted tumour lines. They confirmed the range of responsiveness and tendency towards individuality of the growth delay data. Cell survival in most cases was exponentially related to drug dose. This compilation of a large amount of experimental data supports the belief that human tumour xenografts broadly maintain the level of chemotherapeutic responsiveness of the source tumours in man.

During the past seven years we have coordinated a substantial programme of work on human tumour xenografts in immuno-suppressed mice. Much of the work was performed by clinical scientists who were released from clinical work to pursue a 1–2 year period of laboratory research; their names are listed below.* Each project concentrated upon a specific category of human cancer and led to its own specific conclusions. The purpose of this review is to bring together the therapeutic results of all these investigations and to present such general conclusions as can be drawn at the present time. These conclusions relate to two main questions: How satisfactory are human tumour xenografts for the evaluation of therapeutic response? and, To what extent do the results correspond with clinical experience in chemotherapy?

### Immuno-suppressed mice

The earliest studies in this series of experiments were performed using CBA mice that were immuno-suppressed by 9 Gy whole-body radiation followed immediately by a bone marrow graft (Kopper & Steel, 1975; Nowak *et al.*, 1978). The conventional procedure involved a graft of $5 \times 10^6$ nucleated marrow cells for reconstitution. Attempts to improve the receptivity of the mice to human tumour grafts indicated that some improvement was gained by reducing the size of the marrow graft to $2 \times 10^5$ cells and the mice survived equally well. This procedure was not extensively used, however, because of the development of a simpler and probably more effective procedure.

It has been observed (Millar *et al.*, 1978) that a number of cytotoxic drugs, given to mice a few days before whole-body radiation, increase the dose of radiation that can be tolerated. Increased radiation resistance is found both in the bone marrow and in the intestinal epithelium (Phelps & Blackett, 1979). Among the various drugs that produce this effect, cytosine arabinoside (Ara C) is particularly useful, since it produces a good degree of protection and is well tolerated in single doses. The optimum procedure is to give $200 \, mg \, kg^{-1}$ i.p. 48 h before irradiation. Steel *et al.*, (1978) found that this allowed mice to tolerate 9 Gy whole-body irradiation without the need of a bone marrow graft; cell titration tests using cells from a xenografted human pancreatic carcinoma showed that mice prepared in this way were more receptive to grafting than marrow-reconstituted mice or even than nude mice. This procedure has been standard in this Department since that time.

*M.J. Bailey, R. Berman, A.C. Jones, L. Kopper (Budapest), K. Nowak, D. Raghavan (Melbourne), A.Y. Rostom, P.J. Selby, A.J. Shorthouse, J.M. Thomas, E.A. Wist (Oslo).

Received 19 August 1982; accepted 23 September 1982.

0007-0920/83/010001-13 $01.00

© The Macmillan Press Ltd 1983.

An important disadvantage of immuno-suppressed mice is their tendency to recover the ability to reject human tumour cells during the few months following the procedure. Steel *et al.*, (1978) found evidence for this by 6 weeks and it was subsequently found that raising the dose of whole-body irradiation increased the length of time for which the mice seemed highly receptive to about 3 months (Steel *et al.*, 1980). Mice given radiation doses of 10 or 11 Gy are more fragile than those given the standard dose and the oral administration of neomycin was necessary to reduce mortality.

Further attempts to improve the receptivity of mice to xenografts were reported by Bailey *et al.*, (1980*a*). In an effort to improve the take-rate of human breast tumours, which have widely been found difficult to graft, a variety of modifications were made to the immuno-suppression procedure. The age of the recipients at thymectomy was reduced from 4 to 3 weeks; the radiation dose was raised to 10 Gy, silica particles were injected i.p., oestrogen was administered, castrated male mice were used, the size of the tumour implant was increased, and nude mice were also used (these procedures were, of course, only tested singly). None of the techniques appeared to be an improvement over the standard procedure and the overall success rate (8 lines established from 80 patients) remained comparable with results reported by other investigators (Giovanella *et al.*, 1976; Sebesteny *et al.*, 1979; Rae-Venter & Reid, 1980).

### Success rate in establishing xenograft lines

During the course of the present programme a total of 329 individual human tumours have been investigated. Table 1 shows their breakdown by tumour type. The main emphasis has been on colo-rectal tumours (52% success rate), breast carcinomas (10%), bronchial carcinomas (78%), testicular teratomas (30%) and ovarian carcinomas (23%). These success rates indicate the proportion of clinical specimens that yielded serially transplantable lines. It must be emphasised that each of these tumour types was studied by a different scientist and that, as indicated in the previous section, the technique of immune deprivation has varied over the 7-year period. It would, however, seem clear that breast and ovarian tumours (especially ascites) have generally been more difficult to graft, with melanomas and tumours of the colon and bronchus being relatively successful.

**Table I** Summary of xenograft success rates

| *Tumour location and type* | *Number of patients tested* | *Number of lines established* | *Success rate (%)* | *References* |
|---|---|---|---|---|
| Colon-rectum ca. | 65 | 34 | 52 | Pickard *et al.*, (1975); Nowak *et al.*, (1978) |
| Stomach ca. | 1 | 0 | | |
| Pancreas ca. | 2 | 1 | 50 | |
| Glioma | 5 | 0 | | |
| Melanoma | 15 | 9 | 60 | Selby *et al.*, (1980) |
| Breast ca. | 80 | 8 | 10 | Bailey *et al.*, (1980*a*) |
| Bronchus: adenocarcinoma | 6 | 6 | 100 | |
| squamous | 17 | 11 | 65 | Shorthouse (1980*a,b*) |
| small-cell | 18 | 15 | 83 | |
| large-cell | 8 | 6 | 75 | |
| Testicular tumours: MTU | 26 | 9 | 35 | |
| MTI | 17 | 3 | 18 | |
| MTT | 6 | 2 | 30 | Gibbs *et al.*, (unpublished), referred to in Raghavan *et al.*, (1981) |
| seminoma | 2 | 0 | | |
| mixed | 8 | 3 | 38 | |
| Ovary ca.: solid | 44 | 11 | 23 | Selby *et al.*, (1980); Jones (unpublished) |
| ascites | 9 | 0 | | |
| | 329 | 118 | 36 | |

### Maintenance of biological characteristics in xenografts

Over the course of this series of projects a considerable body of evidence has accumulated on the extent to which xenografts maintain the characteristics of the source tumours. A basic requirement is the maintenance of a human karyotype. This was studied in detail by Reeves & Houghton on 2 colon carcinomas which formed part of a related research project. Human karyotypes were retained through serial passage although there were progressive changes in the distribution of chromosomes. An extra chromosome 19 appeared in one tumour and the progressive selection of sidelines was noted. Human chromosomes were also found in the 2 colonic and bronchial xenografts studied by Kopper & Steel (1975), in 10 melanomas studied by Selby *et al.*, (1980), in 16 bronchial carcinomas studied by Shorthouse *et al.*, (1980), in 9 ovarian carcinomas (Jones *et al.*, 1982) and in 6 testicular teratomas (Raghavan *et al.*, 1981). These investigations (which were performed by J. Mills and G. Casey) showed the occasional occurrence of a mouse mitotic figure which could arise from proliferating cells in the mouse stroma. No hybrid karyotypes were ever observed. There was, however, one case of a rapidly-growing tumour with an exclusively mouse karyotype appearing at the transplantation site of a teratoma xenograft which originally contained human chromosomes. Studies of LDH isoenzymes (Houghton & Taylor, 1978*a*) similarly confirmed the human origin of 6 colon carcinoma xenografts and all of 7 ovarian carcinoma lines were positive for human epithelial membrane antigen (Jones *et al.*, 1982).

Histological comparisons of the xenografts with their source tumours have been made in each type of tumour. In agreement with other investigators, our experience has been that the histological characteristics of the source tumours are usually well maintained by xenografts. Often the stromal elements are less pronounced and this may be associated with the more rapid growth of the tumours in the mouse than in man, and with the fact that the stromal tissue is of mouse origin (Warenius, 1980). There was a frequent, but not universal tendency for the xenografts to be more anaplastic and pleomorphic than the source tumours. Houghton & Taylor (1978*a*) in colonic carcinomas and Shorthouse (1981) in bronchial carcinomas found that the pattern of production of epithelial mucins was well maintained. We have not observed the tendency towards increased differentiation reported by Sharkey *et al.*, (1978).

Tests for the presence of marker substances have been made in a number of disease categories. Shorthouse *et al.*, (1982) demonstrated positive assays for ectopic hormones in bronchial carcinomas: calcitonin (in 13% of xenografts), βHCG (in 13%), ACTH (in 78%) and CEA (in 56%). The synthesis (as opposed to presence) of calcitonin, βHCG and ACTH was also demonstrated in proliferative cultures of a few cell lines and in 2 of these cases βHCG was also demonstrable in mouse plasma. Marker production in teratoma xenografts has been summarised by Raghaven *et al.*, (1981). Among 6 lines that were studied, 2 showed strong production of alphafoetoprotein (AFP) and in both cases the original patient specimen was also strongly positive for AFP. Similarly, a negative assay correlated with negative in the original specimen. The production of CEA by colon carcinoma xenografts was studied by Houghton & Taylor (1978*a*). The protein was detected in all tested specimens of xenografts and high levels were associated with high levels in the clinical specimens. When affinity-purified antibodies to CEA were injected into mice bearing breast carcinoma xenografts (3 of the lines indicated in Table I) specific localisation in tumour tissue was observed (Moshakis *et al.*, 1981).

### Growth rates

The growth rates of xenografts were determined by caliper measurements on s.c. tumour implants. In most cases these were transformed into tumour volumes using the equation for the volume of an ellipsoid but in the case of the teratomas a calibration curve technique was used in which an empirically-determined relationship between the caliper measurements and the excised tumour weight was used to interpret subsequent caliper measurements (Steel, 1977, p.6).

A summary of growth rate data is given in Table II. The measurements were usually made on tumours in the size range 0.1–0.5 g (about 8–13 mm diameter). Growth rate often decreased with increasing tumour size, as is almost always found with syngeneic or xenografted tumours in the mouse (Steel, 1977). It can be seen that in passage 0 (man–mouse) the average volume-doubling times range from 13.5 days in melanomas to 29.5 days in breast carcinomas. This difference is just significant at the 5% level. In the subsequent passages an increase in growth rate was found in all the tumour types, the overall average doubling time falling from 19.8 days to 11.1 days. Further acceleration sometimes occurred beyond passage 4. The most detailed study of growth acceleration through

**Table II** Volume-doubling times of xenografts

| | *Passage 0** | | | *Passage 2–4** | | | |
|---|---|---|---|---|---|---|---|
| | *Mean* | *SD* | *n* | *Mean* | *s.d.* | *n* | *References* |
| Colon ca. | | | | 14.5 | 6.0 | 22 | Kopper & Steel (1975); Nowak *et al.*, (1978) |
| Colon ca. | 22.3 | 13.0 | 5 | 12.1 | 4.1 | 5 | Houghton & Taylor (1978*b*) |
| Breast ca. | 29.5 | 17.5 | 8 | 9.5 | 3.0 | 8 | Bailey *et al.*, (1980*a*) |
| Lung ca.: | | | | | | | |
| Small-cell | 21.2 | 14.8 | 13 | 14.0 | 10.4 | 7 | Shorthouse *et al.*, (1980*a* and *b*) Shorthouse (1981) |
| Squamous | 17.8 | 7.1 | 10 | 10.7 | | 2 | |
| Adenocarcinoma | 15.0 | 6.2 | 6 | 9.4 | 6.0 | 4 | |
| Large-cell | 14.4 | 15.0 | 6 | | | 2 | |
| Melanoma | 13.5 | 9.2 | 7 | 12.0 | 8.0 | 8 | Selby *et al.*, (1980) |
| Ovarian ca. | 21.5 | 13.0 | 12 | | | | Selby *et al.*, (1980); Jones (unpub.) |
| Teratoma | | | | 8.1** | | | |
| | | | | | 1.4 | 4 | Gibbs *et al.*, (unpub.) |

Volume-doubling times in days.
*Passage 0 is the man–mouse passage;
Passage 1 is the first mouse–mouse passage etc.
**Passages 3–10.

sequential passage was the work of Houghton & Taylor (1978*b*) who studied 6 colon carcinomas through up to 10 passages in male and female mice; the growth-rate changes were most marked during the first 3 passages. It will be seen in Table II that acceleration may not occur proportionately in each tumour type. For instance breast carcinomas, which grew the slowest in the first passage, grew faster than other tumours in the subsequent passages.

It is of some interest to ask whether there is a correlation between the success rate of xenografts in the first passage and their growth rate. Are we selecting tumours that grow rapidly? A comparison of the data in Tables I and II reveals no correlation between the growth rate and success rate of these major xenograft categories (Figure 1). The only result that is suggestive of such a correlation is the low success rate and slow growth of the breast carcinomas. However, a positive correlation between growth rate and take-rate among bronchial carcinoma xenografts was found by Shorthouse (1981). Selection in favour of rapidly growing cells is suggested by the clear tendency of xenografts to accelerate during passage in mice and by their tendency towards higher growth rate than is found in the patients. Clinical tumour growth rate data are available on few of the patients who donated tissues for the xenografts in this series (Shorthouse, 1981) but it is possible to compare the present data with other published data on human tumour growth rates (Steel, 1977; Table 1.2 and Figure 1.19). Lung metastases from carcinomas in various

**Figure 1** Lack of correlation between success-rate and growth rate. Success-rates (Table 1) indicate the proportion of tested patients from whom transplantable lines were obtained; growth rates (Table 2) are for passage 0 (man–mouse) of those lines that grew.

primary sites have been found to have a wide range of growth rates whose average is around 60–90 days (not significantly different among the primary sites). It would therefore seem that on average the xenografts that do grow in mice have, even in their earliest passage, a higher growth rate than is found in most human lung metastases. The difference could be approximately a factor of 2 to 6.

### The *in situ* response of xenografts to chemotherapy

Much of the work in the present programme on the response of xenografts to chemotherapeutic treatment has employed tumour growth delay as the end-point of effect. A group of immunosuppressed mice are implanted with tumours, usually within a few days of the immunosuppressive radiation treatment. Not all implants grow at the same rate or with the same latency period. When a good number of them have grown beyond about 7 mm diameter the tumours are accurately measured. Those falling within a defined range of size (say 8–10 mm average superficial diameter) are selected and split into comparable groups each containing, say, 6–10 tumours. One group is kept as a control, the others are treated with one or more drugs at one or more dose levels. Caliper measurements are then continued approximately 3 times per week and tumour volumes are calculated either by a geometrical formula or using a calibration curve technique.

In an idealised situation the results are as shown in Figure 2. Growth of the treated tumours is retarded and they may even temporarily regress. The response curve for the treated tumours may be divided into 2 components: a declining component that represents the clearance of dead tissue and a regrowth component that initially is hidden but which eventually becomes dominant (see Steel, 1977, p. 249). It is the second of these that reliably indicates the effects of treatment. The usual procedure, therefore is to choose a tumour volume that is a simple multiple of the tumour size at treatment and to measure the time taken for the treated and control tumours to reach this size. The greater the multiple, the more confident one can be that the treated tumours are actively regrowing beyond the treatment size.

**Figure 2** Diagram of tumour volume response to treatment, indicating the measurement of tumour growth delay and fractional volume reduction (FVR).

If, for simplicity, the end-point is taken at a relative volume of twice the treatment size then we can determine the time taken ($T_2$, $T'_2$) for control and treated tumours to double their volume. The difference ($T'_2 - T_2$) equals the actual tumour growth delay.

If it is necessary to compare growth delay values between tumour types that differ widely in growth rate, the actual growth delay is clearly unsatisfactory. We have therefore followed the practice of calculating

$$\text{Specific Growth Delay} = \frac{T'_2 - T_2}{T_2}$$

This could be described as the number of volume-doubling times by which growth is delayed and it allows comparisons to be made between tumours of different growth rate. Nevertheless, we recognise that even this manoeuvre is not ideal. There is good evidence (Stephens & Peacock, 1977; Steel & Stephens, 1978) that the tumour cells which survive cytotoxic treatment can repopulate with a doubling time that is considerably shorter than the doubling time of tumours at the time of treatment. This work also shows from one cytotoxic drug to another the rate of repopulation may differ. To interpret or to compare growth delay values one would like to divide them by the average doubling time of surviving clonogenic cells during the regrowth period up to $T'_2$ but of course this is not known. The specific growth delay values as calculated must be regarded as the best approximation available to a standardised growth delay estimate.

The choice of drug dose is another difficult problem in xenograft chemotherapy studies, especially where the intention is to compare the effectiveness of a series of drugs. The procedure used throughout the present group of studies has been to employ each drug at its maximum tolerated dose in mice. The rationale behind this lies in the claim by Freireich *et al.*, (1966) that a linear relationship exists between the $LD_{10}$ doses of cytotoxic drugs in mice and their maximum-tolerated levels in man. The necessary comparative pharmacokinetic data between man and mouse that might allow a more reliable choice of doses are not available.

Figure 3 is a summary of growth delay data on 6 types of human cancer grown as xenografts. In each case the specific growth delay at an $LD_{10}$ drug dose has been calculated. The actual drug doses used varied slightly from one study to another and the teratomas and small-cell lung tumours were so sensitive to treatment that in these cases to avoid cures it was necessary to stay well below the $LD_{10}$ level. The growth delay values have therefore been

**Figure 3** Summary of xenograft response to chemotherapy. The ordinate in each panel indicates the specific growth delay (volume doubling times saved by $LD_{10}$ single drug dose). Each point indicates a separate xenograft line; upward arrow indicates more than half the grafts failed to regrow. The histograms show the mean growth delay for each group of lines, and is not calculated when the spread of values is clearly not Gaussian. Drug abbreviations given in Table III. Drug combinations are: CMF(CY+MTX+FU); AV(Adr+VCR); MCC(MTX+CY+CNU); VAP(VCR+Adr+Pro); CAF(CY+Adr+FU).

adjusted to the $LD_{10}$ dose by linear extrapolation or interpolation and the $LD_{10}$ values used for this are given in Table 3. The choice of drugs used against each tumour was influenced by their known clinical activity or potential interest. The panels of data in Figure 3 are arranged in order of decreasing tumour response. Colo-rectal carcinomas, non-small-cell lung tumours and melanomas did not significantly exceed an average specific growth delay of 1.0 with any drug. Breast carcinomas responded well to 4 of the single agents as well as to the 2 drug combinations. Small-cell lung tumours were cured by large doses of cyclophosphamide, procarbazine, or the MCC combination (methotrexate, cyclophosphamide, CCNU). The testicular tumours were similarly very responsive: the growth delay values given have in most cases been extrapolated from results actually obtained with doses around one-fifth of the $LD_{10}$ level.

It is apparent that these data broadly reflect clinical experience with chemotherapy in these disease areas. The implications of this will be considered in more detail under **General conclusions**.

### Volume reduction as an alternative *in situ* end-point

A serious potential problem in xenograft growth delay experiments arises from the fact that the greater the magnitude of the therapeutic response the longer host defence mechanisms have to act. In so far as the host is capable of being immunised and of mounting an immunological attack on the tumour, this could lead to a systematic enhancement of tumour response which may be dose-dependent. The participation of host immune

**Table III** Assumed $LD_{10}$ dose levels

| *Drug* | *Abbreviation* | $LD_{10}\,(mg\,kg^{-1})$ |
|---|---|---|
| Cyclophosphamide | CY | 250 |
| Melphalan | Mel | 15 |
| *cis*-Platinum | Pt | 10 |
| CBDCA | CB | 160 |
| Hexamethylmelamine | HMM | 350 |
| CCNU | CNU | 50 |
| MeCCNU | MNU | 35 |
| Procarbazine | Pro | 1500* |
| Bleomycin | Ble | 300 |
| Adriamycin | Adr | 12 |
| Actinomycin D | AD | 0.5 |
| 5-Fluorouracil | FU | 185 |
| Methotrexate | MTX | 100* |
| Vinblastine | VLB | 2 |
| Vincristine | VCR | 1.8* |
| DTIC | | 200 |
| VP16 | VP | 40* |
| Mitomycin C | MC | 6.5 |
| Streptozotocin | SZ | 250 |

*All drugs given as a single i.p. dose, except MTX which was usually given in 3 doses over 24 h, procarbazine given in 5 daily doses, vincristine on days 1 and 5, and VP16 in 3 daily doses.

response in enhancing tumour cure has been well demonstrated in the non-xenograft situation (Mantovani *et al.*, 1979; Hengst *et al.*, 1981). In a number of chemotherapy studies on xenografts tumour cure was observed at drug doses that on the basis of lower dose studies would not have been expected to produce a very long growth delay. For instance, Kopper & Steel (1975) using a small-cell bronchial carcinoma in marrow-reconstituted mice found that cyclophosphamide gave a growth delay of 1.1 volume-doubling times at a dose of $120\,mg\,kg^{-1}$ and 3/8 tumours failed to regrow after a dose of $180\,mg\,kg^{-1}$. Such evidence suggests that clonogenic cell kill of only perhaps 2 decades (a surviving fraction of 1%) may achieve substantial tumour control when combined with an effective immune response. Whether this also leads to upward-bending dose-response curves at noncurative doses is not yet clear, for a survey of the literature reveals few well-defined dose-response curves for xenografts. Two studies from the present programme have examined tumour growth delay as a function of dose and in both cases the dose-response curves were almost linear: small-cell lung tumours studied by Shorthouse *et al.*, (1982) and breast carcinoma xenografts studied by Bailey *et al.*, (1980*b*). However, a survey of published dose-response data for the radiation treatment of xenografts shows that there has been a tendency towards upward curvature (see e.g. Rofstad & Brustad, (1978, 1980), who used melanomas and osteosarcomas in nude mice).

In face of the evident possibility of artefacts resulting from this phenomenon, we have also explored the use of a tumour growth inhibition end-point: at some fixed time after treatment to measure the relative volume of treated and control tumours. This procedure has been widely used in experimental chemotherapy, including some studies on xenografts (Povlsen & Jacobsen, 1975; Shimosato *et al.*, 1977). The method has been criticised on the grounds that if the size determinations are made too early tumour regression will not yet be complete and the magnitude of response will be underestimated. But the method could be reliable provided there is evidence that at the time of measurement the treated tumours are actively regrowing. We have therefore re-analysed our data using this restriction, evaluating for each treated and control tumour the relative tumour size ($V$) at the end of the growth period compared with their size at the time of treatment and then calculating

$$\text{Fractional Volume Reduction (FVR)} = \log_{10}\frac{\text{mean } Vc}{\text{mean } Vt}$$

(see Figure 2).

This gives the number of decades by which the treated tumour volume is reduced below the control value. FVR = 1.0 indicates treated tumours 1/10 of control; FVR = 2.0 indicates treated tumours 1/100 of control.

Results on a range of different xenograft types are shown in Figure 4 as a comparison with the corresponding growth delay value. For breast,

**Figure 4** Correlation between FVR and growth delay. □ Small-cell ca.; ▽ Non-small-cell ca. bronchus; ○ Breast ca.; △ Colo-rectal ca.; ● Testicular teratoma.

colon and lung tumours (small-cell and non-small-cell tumours) the data seem to fall around a common line; for testicular teratomas the data give a steeper slope. This could be due to the rapid regression of the teratomas making it easier to obtain an FVR determination that truly reflects the reduction in the volume of viable tissue. The slope of the curve for teratomas is about 0.3, close to $\log_{10}2$ and this is consistent with the expectation that a delay of one volume-doubling time results in treated tumours that are half the size of controls.

The FVR method is now in use for therapeutic studies on xenografts, for instance in teratomas which tend, especially after treatment, to become cystic. The opportunity to excise the tumours at one particular time and determine the cellular composition by weighing is an associated advantage over the growth delay method.

### Comparative cell survival studies among xenografts

The principal alternative to *in situ* methods of measuring therapeutic response in xenografts is to determine clonogenic cell survival. Tumour-bearing mice are treated, the tumours are excised within about a day, disaggregated into a single-cell suspension and the cells placed in an *in vitro* (or perhaps *in vivo*) environment in which their ability to form a colony can be evaluated (for review see Elkind & Whitmore, 1967; Steel, 1977). A number of clonogenic assays have been developed for xenografted human tumours (Courtenay & Mills, 1978; Smith *et al.*, 1976; Selby *et al.*, 1980; Selby & Steel, 1982). Plating efficiencies vary widely from very low levels in some tumours to $\sim$ 30% in favourable cases. Problems of tissue disaggregation are sometimes severe and at the present time only a minority of xenografted tumours are amenable to reliable clonogenic assay.

The great advantage of clonogenic cell survival as an end-point for xenograft studies is that by removing the treated and control cells into a common environment problems of host defence mechanisms are avoided. Other advantages of this approach are its sensitivity (often down to a surviving fraction of $10^{-3}$) and its ability to detect the presence or absence of resistant components within the tumour or a "shoulder" at low drug doses.

Chemotherapy studies of cell survival in the present research programme have included studies on pancreatic and bronchial carcinomas, melanomas, and a single survival curve for a colon carcinoma. Survival curves were in almost all cases indistinguishable from an exponential, with rare examples of a small shoulder. The only marked exception to this was in the response of two of the melanomas to DTIC, where the curves fell to a clear plateau at about $10^{-2}$ survival for drug doses in excess of $100\,mg\,kg^{-1}$. The steepness of the survival curves can therefore in most cases be expressed by the surviving fraction at the $LD_{10}$ dose and these values (given as decades of cell kill) are summarised in Table IV. Values in excess of 3.0 indicate situations in which the survival at the $LD_{10}$ dose was too low to measure, and linear extrapolation has been used.

Although data in Table IV do not include a wide variety of drugs or an extensive range of tumour types they do support the implications of the growth delay data that among a group of similar tumours the effectiveness of a particular drug can vary widely. It is even possible in some cases to quantify the variation: among the melanomas there is a 3-fold range of sensitivity to melphalan and a $>$5-fold range for MeCCNU. The two pancreatic tumour lines have very similar sensitivities to most drugs used but a $>$5-fold difference for hexamethylmelamine (Courtenay *et al.*, 1982). There is little opportunity to ask whether the cloning data also support the large differences between sensitive and resistant tumour types seen in Figure 3, for only 2 data points are available for nominally chemosensitive disease: the small-cell carcinomas treated with the 3-drug combination MCC. These two were, however, very responsive.

### Relation between cell survival and tumour growth delay

It is of some interest to enquire to what extent measurements of clonogenic cell survival correlate with growth delay measurements on the same drugs and tumours. The number of tumours in which this comparison can be made is not large and the data are summarised in Figure 5. For points below a surviving fraction of $10^{-3}$ the survivals have been obtained by extrapolation up to the dose used in the growth delay determinations.

Among the sensitive tumours (i.e. those that give a specific growth delay greater than 1.0 or a surviving fraction below say 0.3) there is a general tendency for growth delay and log cell kill to increase together. There is by no means a tight relationship between these parameters, nor would one expect this. As shown in the B16 mouse melanoma by Stephens & Peacock (1977) the growth delay achieved for a fixed level of cell kill depends upon the drug used: for cyclophosphamide the growth delay was considerably greater than for CCNU and this difference was attributed to a faster rate of repopulation by surviving clonogenic cells after CCNU.

**Table IV** Cell survival following *in vivo* treatment (Decades of cell kill at $LD_{10}$ dose)*

| | *PE*** | *CY* | *Mel* | *MNU* | *Adr* | *DTIC* | *HMM* | *Pt* | *FU* | *MC* | *SZ* | *MCC*‡ |
|---|---|---|---|---|---|---|---|---|---|---|---|---|
| Pancreatic ca.: | | | | | | | | | | | | |
| HX32 | 25–30 | 0.4 | 3.0 | 1.0 | | | 2.3 | 0.7 | | 3.5 | 0 | |
| HX58 | 7–18 | 1.0 | 3.0 | 1.0 | | | 0.2 | 0.7 | | | 0 | |
| Melanomas: | | | | | | | | | | | | |
| HX34 | 10–50 | 0.4 | 2.2 | 7 | 0.1 | 2.6† | 1.5 | 0.9 | | 0.9 | 2.3 | |
| HX40 | 2–13 | | 2.1 | 3.0 | 0.1 | | | | | | | |
| HX41 | 10–75 | 0.3 | 0.9 | 0.3 | 0 | 0.1 | | | | | | |
| HX46 | 2–6 | | 3.0 | 2.0 | 0.1 | | | | | | | |
| HX47 | 3–18 | | 2.0 | 4.5 | 0 | 2.2† | | | | | | |
| Colon ca.: | | | | | | | | | | | | |
| HX18 | 1.0 | 0.6 | | | | | | | | | | |
| Small-cell lung tumours: | | | | | | | | | | | | |
| HX72 | 3 | | | | | | | | | | | 4.3 |
| HX81 | 3–6 | | | | | | | | | | | 21 |
| Lung Adenocarcinoma: | | | | | | | | | | | | |
| HX70 | 2–5 | 0.7 | | | 0.5 | | | | 0.5 | | | |

*Values in excess of 3.0 were obtained by extrapolation. Results on melanomas mostly determined by the agar diffusion chamber assay (Selby *et al.*, 1980); the rest by *in vitro* assay (Courtenay & Mills, 1978).
**Plating Efficiency (%). Abbreviations for drugs are given in Table III, together with assumed $LD_{10}$ values.
†Non-exponential survival curves.
‡MCC = methotrexate + CY + CCNU.

**Figure 5** Correlation between specific growth delay and surviving fraction. Full symbols, bronchial carcinomas (● adenocarcinoma treated with CY, Adr or FU; ▲ small-cell ca. treated with MCC). Open symbols, melanomas (□ Mel, △ MNU, ▽ DTIC, ○ Adr). The broken lines show the expected relation when the average doubling time for repopulation is a factor 2 or 10 times less than the untreated volume-doubling time.

If we assume that the scatter of the data in this chart is attributable to differences in the amount of acceleration in tumour cell repopulation that occurs during regrowth it is possible to calculate how wide this difference must be. To encompass almost all the points the ratio of the post- to pre-treatment growth rates must range from about 2 to 10.

### Evidence for individuality among human tumours

The data presented in Figure 3 and Table IV clearly show that xenografts generally maintain the chemotherapy response characteristics of the class of tumours from which they are derived. A related question is what variations in chemosensitivity exist among a group of xenografts of the same class. Do they appear as a generally high or low sensitivity to drug treatment or are there clear differences in the *spectrum* of drug response from one xenograft to another? This is an important question because it relates to the whole concept of individualised chemotherapy for cancer (Hamburger, 1981).

Evidence for individuality among xenografts has come from a number of the studies that make up the present programme:

1. Nowak *et al.*, (1978). Ten lines of colo-rectal carcinoma were challenged with up to 8 single agents and evaluated by tumour growth delay. The overall average specific growth delay was only 0.5 but 14 of the 68 determinations exceeded 1.0 and 5 exceeded 2.0 doubling times. These 5 "good" responses were scattered among all the 10 tumour lines and among 6 of the 8

drugs. Even drugs such as methotrexate and actinomycin D, which ranked poorest overall, achieved good responses (2.7 and 2.9 in one tumour line.

2. Bailey *et al.* (1980*b*). This was a similar study in breast carcinoma xenografts, employing 5 tumour lines challenged with 6 single agents and two drug combinations. By comparison with the colo-rectal tumours, this disease was more responsive to chemotherapy with an overall mean specific growth delay of 2.0. In this case there were significant differences among the tumour lines in their response to chemotherapy as well as significant differences among drugs in their effectiveness. Superimposed on these differences was an apparent degree of individuality in response to the most effective drugs: of the 5 tumour lines, one responded best to the CMF combination, 2 were best with melphalan and 2 best with the AV combination (see Legend to Figure 3).
3. Shorthouse *et al.*, (1980*ab*; 1982). In this extensive study on xenografts of bronchial carcinomas there was evidence that in addition to the marked differences between small-cell and non-small-cell tumours, those within a particular histopathological type also differed in drug sensitivity. There was no evidence for a difference in *spectrum* of drug response but some tumours were markedly more responsive than others.
4. Selby *et al.*, (1980). In this cell survival study on 5 melanoma xenograft lines measurable responses were only available to 3 drugs (melphalan, MeCCNU and DTIC). There was no evidence for individuality among these tumours and drugs. However, a parallel programme of *in vitro* chemosensitivity testing was carried out by Bateman *et al.*, (1980) and a statistical analysis of the data yielded evidence for significant individuality in the response of 5 melanoma xenografts to melphalan, MeCCNU, *cis*-platinum and adriamycin.
5. Courtenay *et al.*, (1982). Two pancreatic carcinoma xenografts were evaluated in response to 6 cytotoxic drugs by clonogenic cell survival. Their response to 4 agents (melphalan, MeCCNU, *cis*-platinum and streptozotocin) was indistinguishable. There was a slight difference in response to cyclophosphamide but a large difference in response to hexamethylmelamine.

These studies comprise a body of data that broadly supports the individuality hypothesis. In the case of the growth delay data the evidence would have been stronger if it had been possible to perform a series of repeat experiments with each tumour-drug combination. But in the case of the cell survival studies (especially no. 5 above) where repeat experiments were performed the difference in spectrum of drug response among the tumours was highly significant.

**Comparison of xenograft with donor patient response**

The most precise and relevant way of validating the xenograft system for therapeutic purposes is directly to compare xenograft response with the clinical response of the donor patient. This is logistically a difficult type of investigation because of the combined effect of unavoidable factors: the proportion of donor patients who receive chemotherapy may be small; few of them may give an objectively measurable clinical response; the xenograft success rate may be <50%. In spite of these limitations, comparisons of this type have been possible in 3 disease areas:

1. Colo-rectal Cancer. Four of the patients studied by Nowak *et al.*, (1978) received chemotherapy (MeCCNU, 5FU and DTIC). In three there was no evidence of a therapeutic response and the disease progressed. One achieved an objective clinical response, and the xenografts from this patient were the most responsive (of the 10 xenograft lines studied) to MeCCNU and also responsive to 5FU.
2. Melanoma. Two of the donor patients in the melanoma study of Selby *et al.*, (1980) had measurable lung metastases and were treated with melphalan. One (donor of HX47) showed an objective response but the other (HX41) did not. The melphalan cell survival curve for the xenografts was twice as steep in HX47 as in HX41.
3. Bronchial Carcinoma. The major study of this type was that of Shorthouse *et al.*, (1980*ab*). Xenograft and donor patient response was evaluated in 7 small-cell carcinomas, 3 squamous carcinomas, 4 adenocarcinomas and one large-cell carcinoma. The clinical response of small-cell tumours was reflected in highly responsive xenografts, as was the lack of response of the non-small-cell tumours. Two previously-treated patients with small-cell cancer failed to achieve a clinical response, and the xenografts were similarly resistant.

The consistency of agreement between patient and xenograft responses in these studies is encouraging. This does not of course mean that there is a realistic prospect of using xenografts for clinical chemosensitivity testing, since as discussed by Bailey *et al.*, (1981) the limitations of speed of assay and of success rate would limit the proportion of patients helped to only 10–20%.

However, the use of xenografts as models of particular human cancers is supported.

### General conclusions

The body of data reviewed here comes from work on a wide range of types of human cancer, not only in respect of tissue origin but also of responsiveness to cytotoxic drug treatment. The drawbacks of *in situ* growth delay or cell survival studies have been indicated, but in spite of these there is clear evidence that the xenografts respond to drug treatment in a way that broadly would be expected on the basis of clinical experience. In terms of average response to the 3 agents that in each case were most effective, the 6 categories of disease for which results are shown in Figure 3 can be ranked (Table V). This ranking is compared in Table V with estimates of complete response rate achieved in current clinical practice. Response rates vary considerably from one report to another, but we have included in Table V average values from recent review papers or, where these are unavailable, typical values from recent large series. Although the drugs and drug schedules used in these clinical studies were not the same as those used to calculate the xenograft average values, the correspondence between the xenograft and clinical response data is remarkable.

In addition to this plausible inter-type ranking there is also evidence that in any particular tumour type the drugs that do well clinically also rank high in xenografts: cyclophosphamide, CCNU and vincristine in small-cell lung cancer; bleomycin, and *cis*-platinum in testicular teratoma. However, there are also some surprising results, notably the marked effectiveness of procarbazine in small-cell tumours and the relatively poor performance of adriamycin in other than breast tumours. The ineffectiveness of VP16 in teratomas may well be associated with the known difficulty of ensuring high enough concentrations of this agent in a soluble form in mice; we suspect that as a result of this drug delivery problem the present data may under-rate the value of this agent. The relatively poor performance of methotrexate in breast and small-cell tumours is also remarkable, especially in view of the higher growth rate of the xenografts than of the corresponding disease in man. The breast xenografts received single dose treatment, the small-cell xenografts 3 doses over 24 h. We are inclined to accept the xenograft data on methotrexate as possible evidence that the clinical activity of this drug may have been over-rated.

A number of combination chemotherapy schedules have been evaluated in the present work (Kopper & Steel, 1975; Nowak *et al.,* 1978; Bailey *et al.,* 1980*b*; Shorthouse *et al.,* 1982). There has not been space in this publication to review these studies in detail. However, they can be summarised in the statement that in no case was there statistically significant evidence for the growth delay of a drug combination exceeding the aggregate growth delays of the component single agents. Combination chemotherapy was usually effective in that a greater tumour response could be achieved within the toxicity limitations of the host than with any single agent, but this is to be attributed to non-overlapping toxicity rather than to "synergistic" effects against tumour cells.

The assessment of therapeutic response in human tumour xenografts is not a simple matter. *In situ* measurements of response are vulnerable to artefacts from the substantial host response that appears to be mounted against grafts in immuno-suppressed mice. This problem is not restricted to artificially immuno-suppressed mice for there is also evidence for such responses in nude mice (Sordat *et al.,* 1982). Further studies are under way in which

**Table V** Comparative responsiveness to chemotherapy in six categories of human tumour xenograft and their clinical counterparts

| | *Average xenograft growth delay** | *Clinical complete response rate* (%) | *Source of clinical data*** |
|---|---|---|---|
| Testicular teratoma | 5.7 | 70 | Einhorn (1979) |
| Small-cell lung cancer | 4.2 | 31 | Minna *et al.*, (1982) |
| Breast cancer | 1.9 | 15.3 | Hellman *et al.*, (1982) |
| Melanoma | 1.0 | 4 | Wittes *et al.*, (1978) |
| Colo-rectal cancer | 0.76 | 3 | Kemeny *et al.*, (1980) |
| Non-small-cell lung cancer | 0.54 | 5 | Minna *et al.*, (1982) |

*Average number of volume-doubling times saved by maximum tolerated dose treatment using the 3 single agents in common clinical use that were most effective in each category; data from Figure 3.

**These comprise review papers or individual publications that reflect general clinical experience.

this is being investigated, especially in response to local irradiation of the grafts. Clonogenic assays obviate the host response problem but as yet their application to a wide range of xenograft types has been limited by problems of tissue disaggregation and cell cloning. The main benefit of the clonogenic assay work has been in the study of the *shape* of cell survival curves. Drugs were chosen that gave a good response or were of clinical interest for the disease in question. For almost all of these cytotoxic agents the curves have been exponential, implying that the cellular sensitivity was relatively homogeneous within the tumours.

In view of the greater cost and technical limitations a programme on this scale could not have been mounted in nude mice and we are encouraged, in spite of the lack of stability of immune response in immuno-suppressed mice, to continue using them in studies of this type.

As well as acknowledging the participation of the clinical scientists listed above, we are grateful to John Gibbs, Judith Mills and Patricia Wilson for technical support, to Ted Merryweather for care of the animals, and to Louise Parkes for typing the manuscript.

### References

BAILEY, M.J., GAZET, J.-C. & PECKHAM, M.J. (1980*a*). human breast-cancer xenografts in immune-suppressed mice. *Br. J. Cancer*, **42**, 524.

BAILEY, M.J., GAZET, J.-C., SMITH, I.E. & STEEL, G.G. (1980*b*). Chemotherapy of human breast-carcinoma xenografts. *Br. J. Cancer*, **42**, 530.

BAILEY, M., JONES, A., RAGHAVAN, D. & 4 others (1981). Limitation of human tumour xenografts in individual patients drug-sensitivity testing. *Br. J. Cancer*, **43**, 725 (Abstract).

BATEMAN, A.E., SELBY, P.J., STEEL, G.G. & TOWSE, G.D.W. (1980). *In vitro* chemosensitivity tests on xenografted human melanomas. *Br. J. Cancer*, **41**, 189.

COURTENAY, V.D. & MILLS, J. (1978). An *in vitro* colony assay for human tumours grown in immune-suppressed mice and treated *in vivo* with cytotoxic agents. *Br. J. Cancer*, **37**, 261.

COURTENAY, V.D., MILLS, J. & STEEL, G.G. (1982). The spectrum of chemosensitivity of two human pancreatic carcinoma xenografts. *Br. J. Cancer*, **46**, 436.

EINHORN, L.H. (1979). Combination chemotherapy, with *cis*-Dichlorodiammine-platinum (II) in disseminated testicular cancer. *Cancer Treat. Rep.*, **63**, 1659.

ELKIND, M.M. & WHITMORE, G.F. (1967). The radiology of cultured mammalian cells. New York: Gordon & Breach, p. 7.

FREIREICH, E.J., GEHAN, E.A., RALL, D.P., SCHMIDT, L.H. & SKIPPER, H.E. (1966). Quantitative comparison of toxicity of anticancer agents in mouse, rat, hamster, dog, monkey and man. *Cancer Chemother. Rep.*, **50**, 219.

GIOVANELLA, B.C., STEHLIN, J.S., LEE, S.S., SHEPPARD, R. & WILLIAMS, L.J. (1976). Heterotransplantation of human breast carcinomas into nude mice. *Proc. Am. Soc. Cancer Res.*, **17**, 124.

HAMBURGER, A.W. (1981). Use of *in vitro* tests in predictive cancer chemotherapy. *J. Natl Cancer Inst.*, **66**, 981.

HELLMAN, S., HARRIS, J.R. & CANELLOS, G.P. (1982). "Cancer of the breast" In *Cancer: Principles and Practice of Oncology* (Eds. deVita, *et al*,) Philadelphia: Lippincott, p. 914.

HENGST, J.C.D., MOKYR, M.B. & DRAY; S. (1981). Cooperation between cyclophosphamide tumouricidal activity and host antitumour immunity in the cure of mice bearing large MOPC-315 tumours. *Cancer Res.*, **41**, 2163.

HOUGHTON, J.A. & TAYLOR, D.M. (1978*a*). Maintenance of biological and biochemical characteristics of human colorectal tumours during serial passage in immune-deprived mice. *Br. J. Cancer*, **37**, 199.

HOUGHTON, J.A. & TAYLOR, D.M. (1978*b*). Growth characteristics of human colorectal tumours during serial passage in immune-deprived mice. *Br. J. Cancer*, **37**, 213.

JONES, A.C., STRATFROD, I.J., WILSON, P.A. & PECKHAM, M.J. (1982). *In vitro* cytotoxic drug sensitivity testing of human tumour xenografts grown as multi-cellular tumour spheroids. *Br. J. Cancer*, **46**, 000.

KEMENY, N., YAGODA, A., BRAUN, D. & GOLBEY, R. (1980). Therapy for metastatic colorectal carcinoma with a combination of MethylCCNU, 5 fluorouracil, vincristine & streptozotocin (MOP-Strep). *Cancer*, **45**, 876.

KOPPER, L. & STEEL G.G. (1975). The therapeutic response of three human tumor lines maintained in immune-suppressed mice. *Cancer Res.*, **35**, 2704.

MANTOVANI, A., POLENTARUTTI, N., LUINI, W., PERI, G. & SPREAFICO, F. (1979). Role of host defense mechanisms in the antitumor activity of adriamycin and daunomycin in mice. *J. Natl. Cancer Inst.*, **63**, 61.

MILLAR, J.L., BLACKETT, N.M. & HUDSPITH, B.N. (1978). Enhanced post-irradiation recovery of the haemopoietic system in animals pre-treated with a variety of cytotoxic agents. *Cell Tissue Kinet.*, **11**, 543.

MINNA, J.D., HIGGINS, G.A. & GLATSTEIN, E.J. (1982). "Cancer of the breast" in: *Cancer principles and Practice of Oncology* (Eds. deVita, *et al*) Philadelphia: Lippincott, p. 396.

MOSHAKIS, V., BAILEY, M.J., ORMEROD, M.G., WESTWOOD, J.H. & NEVILLE, A.M. (1981). Localization of human breast-carcinoma xenografts using antibodies to carcinoembryonic antigen. *Br. J. Cancer*, **43**, 575.

NOWAK, K., PECKHAM, M.J. & STEEL, G.G. (1978). Variation in response of xenografts of colo-rectal carcinoma to chemotherapy. *Br. J. Cancer*, **37**, 576.

PHELPS, T.A. & BLACKETT, N.M. (1979). Protection of intestinal damage by pretreatment with cytarabine (cytosine arabinoside). Int. J. *Radiat Oncol Biol. Phys.*, **5**, 1617.

PICKARD, R.G., COBB, L.M. & STEEL, G.G. (1975). The growth kinetics of xenografts of human colorectal tumours in immune deprived mice. *Br. J. Cancer*, **31**, 36.

POVLSEN, C.O. & JACOBSEN, G.K. (1975). Chemotherapy of a human malignant melanoma transplanted in the nude mouse. *Cancer Res.*, **35**, 2790.

RAGHAVAN, D., HEYDERMAN, E., GIBBS, J., NEVILLE, A. & PECKHAM, M. (1981). Functional and morphological aspects of human teratoma xenografts. In *Thymusaplastic Nude Mice and Rats in Clinical Oncology*. Stuttgart: Gustav Fischer Verlag.

REA-VENTER, B. & REID, L.M. (1980). Growth of human breast carcinomas in nude mice and subsequent establishment in tissue culture. *Cancer Res.*, **40**, 95.

REEVES, B.R. & HOUGHTON, J.A. (1978). Serial cytogenetic studies of human colonic tumour xenografts. *Br. J. Cancer*, **37**, 612.

ROFSTAD, E.K. & BRUSTAD, T. (1978). The radiosensitizing effect of metronidazole and misonidazole (Ro-07-0582) on a human malignant melanoma grown in the athymic mutant nude mouse. *Br. J. Radiol.*, **51**, 381.

ROFSTAD, E.K. & BRUSTAD, T. (1980). Radiosensitizing effect of misonidazole in acute and fractionated irradiation of a human osteosarcoma xenograft. *Int. J. Radiat. Oncol. Biol. Phys.*, **6**, 1163.

SEBESTENY, A., TAYLOR-PAPADIMITRIOUS, J., CERIANI, R., MILLIS, R., SCHMITT, C. & TREVAN, D. (1979). Primary human breast carcinomas transplantable in the nude mouse. *J. Natl Cancer Inst.*, **63**, 1331.

SELBY, P.J., COURTENAY, V.D., McELWAIN, T.J., PECKHAM, M.J. & STEEL, G.G. (1980). Colony growth and clonogenic cell survival in human melanoma xenografts treated with chemotherapy. *Br. J. Cancer*, **42**, 438.

SELBY, P.J. & STEEL, G.G. (1982). The use of the agar diffusion chamber for the exposure of human tumor cells to drugs. *Cancer res.* (in press).

SHARKEY, F.E., FOGH, J.M., HAJDU, S.I., FITZGERALD, P.J. & FOGH, J. (1978). Experience in surgical pathology with human tumor growth in the nude mouse. In *The Nude Mouse in Experimental and Clinical Research*. p. 187 (Ed. Fogh & Giovanella) New York, Academic Press. p. 187.

SHIMOSATO, Y., KAMEYA, T., KUBOTA, T., HIROHASHI, S., HAYASHI, H., IKEUCHI, S. & NAGAI, K. (1977). Experimental chem-, radio-, and endocrine therapy for human cancers transplanted in nude mice. *Proc. 2nd Int. Workshop on Nude Mice*. Tokyo: University Press.

SHORTHOUSE, A.J. (1981). Experimental chemotherapy of human tumour xenografts. *M.S. Thesis, University of London.*

SHORTHOUSE, A.J., CARTER, S.M. & ELLISON, M.L. (1982). Tumour marker production in human bronchial carcinoma xenografts. *Oncodev. Biol. med.* **3**, 273.

SHORTHOUSE, A.J., JONES, J.M., STEEL, G.G. & PECKHAM, M.J. (1982). Experimental combination and single agent chemotherapy in human lung tumour xenografts. *Br. J. Cancer*, **46**, 35.

SHORTHOUSE, A.J., PECKHAM, M.J., SMYTH, J.F. & STEEL, G.G. (1980*a*). The therapeutic response of bronchial carcinoma xenografts: A direct patient-xenograft comparison. *Br. J. Cancer*, **41**, (Suppl. IV.) 142.

SHORTHOUSE, A.J., SMYTH, J.F., STEEL, G.G., ELLISON, M., MILLS, J. & PECKHAM, M.J. (1980*b*). The human tumour xenograft—a valid model in experimental chemotherapy. *Br. J. Surg.*, **67**, 715.

SMITH, I.E., COURTENAY, V.D. & GORDON, M.Y.(1976). A colony-forming assay for human tumour xenografts using agar in diffusion chambers. *Br. J. Cancer*, **34**, 476.

SORDAT, B.C.M., UEYAMA, Y. & FOGH, J. (1982). Metastases of tumour xenografts in the nude mouse. In *The Nude Mouse in Experimental and Clinical Research. Vol. 2* (Eds. Fogh & Giovanella,) New York: Academic Press. p. 95.

STEEL, G.G. (1977). *The Growth Kinetics of Tumours*. Oxford: University Press.

STEEL, G.G., COURTENAY, V.D., PHELPS, T.A. & PECKHAM, M.J. (1980). The therapeutic response of human tumour xenografts. In *Immunodeficient Animals for Cancer Research*. (Ed. Sparrow.) Macmillan Press. p. 179.

STEEL, G.G., COURTENAY, V.D. & ROSTOM, A.Y. (1978). Improved immune-suppression techniques for the xenografting of human tumours. *Br. J. Cancer*, **37**, 224.

STEEL, G.G. & STEPHENS, T.C. (1978). The relation of cell kinetics to cancer chemotherapy. In *Advances in Pharmacology and Therapeutics. Vol. 10 Chemotherapy*. (Ed. Adolphe) Oxford: Pergamon Press. p. 137.

STEPHENS, T.C. & PEACOCK, J.H. (1977). Tumour volume response, initial cell kill and cellular repopulation in B16 melanoma treated with cyclophosphamide and 1-(2-chloroethyl)-3-cyclophexyl-l-nitrosourea. *Br. J. Cancer*, **36**, 313.

WARENIUS, H.M. (1980). Identification and separation of mouse and human components of heterotransplanted tumours. In *Immunodeficient Animals for Cancer Research*. (Ed. Sparrow) London: Macmillan. p. 207.

WITTES, R.E., WITTES, J.T. & GOLBEY, R.B. (1978). Combination chemotherapy in metastatic malignant melanoma: A randomized study of three DTIC-containing combinations. *Cancer*, **41**, 415.